# ESR Journals editors’ joint statement on Guidelines for the Use of Large Language Models by Authors, Reviewers, and Editors

**DOI:** 10.1186/s13244-023-01600-9

**Published:** 2024-01-24

**Authors:** Bernd Hamm, Luis Marti-Bonmati, Francesco Sardanelli

**Affiliations:** European Society of Radiology (ESR), Vienna, Austria

## Preamble

The impact of artificial intelligence (AI)-assisted technologies, such as Large Language Models (LLMs), chatbots, or image creators, on biomedical publishing was discussed by the editors of radiology journals at the annual Radiology Editors’ Forum, held on August 11–12, 2023, in Chicago, Illinois. The forum was attended by over 40 individuals, representing 30 biomedical imaging journals from 9 countries. In addition to considering the May 2023 ICMJE update [1], the editors considered relevant statements regarding contributions by AI-assisted technologies from other publication committees, associations, and societies, including the World Association of Medical Editors (WAME), the Committee on Publication Ethics (COPE), and the Council of Science Editors (CSE) policies [2–4]. New NIH guidelines to address the role of generative AI-assisted review of submitted applications were also reviewed [5], as well as policies developed by various medical journals and medical publishers [6–12]. At the conclusion of the forum, the following policies were endorsed in principle. With this article, the Editors-in-Chief of the ESR Journals adapt these policies to their journals. It is obvious that generative AI tools will continue to quickly evolve and develop new possibilities in our daily lives. The statements and policies will need to be re-evaluated and updated regularly.

### AI or AI-assisted technologies do not qualify as authors and must not be listed as authors or co-authors [1–3, 6–11]

Nonhuman AI, LLMs, chatbots, machine learning, or similar generative AI technologies do not meet the four ICMJE criteria for authorship. These qualifications were developed to guarantee that all authors accept full responsibility and stand for the integrity of the entire work. Accordingly, only humans can be authors [2]. AI-assisted technologies that were used to generate results should be reported in the article as methodological devices used in the completion of the work, but not included as authors*.*

### Authors must disclose at submission whether they used AI or AI-assisted technologies in their work

Authors who use such technology must clearly describe how AI or AI-assisted technologies were used in the study and/or manuscript preparation. Authors should be transparent when AI-assisted technologies are used and provide information about their use [2, 3]. If the tools were part of carrying out the research and to generate results, authors must provide this information in the Materials and Methods section or in the relevant section of the manuscript (e.g., figure legends for AI-generated figures) [9]. In all cases of use of AI-assisted technologies, authors should include specific details, such as the name and version of the AI tool, date of access, name of the manufacturer/creator [6, 7, 9, 10].

### Authors must disclose at submission whether they used AI or AI-assisted technologies for writing or editing the manuscript

Authors may use LLMs to assist with medical writing and for content editing to effectively communicate their work. These tasks include assistance with medical writing, grammar, language and reporting standards. Authors must transparently report how they used such tools in the writing or editing of their submitted work in the Acknowledgment section. Authors are encouraged to include specific details, such as the name of the language model or tool, version number, and manufacturer [9, 10].

### All authors are fully responsible for any submitted material that includes AI-assisted technologies

AI-assisted technologies cannot distinguish between true and false information. Humans, i.e., the authors, are and remain fully responsible for the submitted manuscript. Authors should carefully review and edit the results of AI-assisted content, because AI can generate authoritative-sounding output that can be biased, incomplete, or partially or completely incorrect [1].

### Authors should be able to assert that there is no plagiarism in their paper, including in text and images produced by AI

Humans must ensure appropriate attribution to all quoted material, including full citations [1]. Authors should acknowledge all sources (including material produced by AI-assisted tools) [2, 6–11]. Authorship attribution requires accountability for the submitted work. Further, authors are responsible for any text generated by an AI-assisted tool in their manuscript (including the accuracy of what is presented and the absence of plagiarism) and for acknowledging all sources (including material produced by the AI-assisted tool) and ensuring the accuracy and completeness of citations [2]. AI-generated material cannot be referenced as primary source [1].

### Any content created by AI or AI-assisted tools must be labelled

The submission and publication of content/images created by AI, language models, machine learning, or similar technologies is discouraged, unless it is part of the formal research design or methods, and is not permitted without clear labelling, meaning a description of the content that was created, the name of the model or tool, version and extension numbers, and manufacturer [6]. Authors are fully responsible for the integrity of the content generated by these models and tools [6]. When generative AI itself is the focus of a study, the use of AI should be explicitly detailed in the Materials and Methods section [9].

### Reviewers and editors are obligated to confidentiality and should not upload manuscripts to software or other AI-assisted tools where confidentiality cannot be assured [1, 2]

Reviewers and editors are trusted and required to maintain confidentiality throughout the manuscript review process. Authors trust the reviewers and editors to protect their proprietary, sensitive, and confidential ideas. The use of AI-assisted tools may violate peer review confidentiality expectations, and several journals have followed the ICJME and WAME guidelines and state that entering any part of the manuscript or abstract or the text of your review into a chatbot, language model, or similar tool is a violation of the journals’ confidentiality agreement [7, 9, 12]. The review process is valued for its human expert perspective and human oversight with decision-making in scholarly publication, including the need for accountability and human oversight [9, 13]. If a reviewer or editor used an AI tool as a resource for his/her review in a way that does not violate the journal’s confidentiality policy, he/she must provide the name of the tool and how it was used.

## Data Availability

All the relevant content is included in this Editorial.

## References

[1] International Committee of Medical Journal Editors. Recommendations. Available at: https://www.icmje.org/icmje-recommendations.pdf. Accessed 1 June 2023 10.12771/emj.2024.e48 PMC12093628 40704003

[2] Zielinski C, Winker MA, Aggarwal R et al (2023) Chatbots, generative AI, and scholarly manuscripts: WAME recommendations on chatbots and generative artificial intelligence in relation to scholarly publications. Available at: https://wame.org/page3.php?id=106. Accessed 1 June 2023 10.25100/cm.v54i3.5868 PMC10712422 38089825

[3] Committee on Publication Ethics. Authorship and AI tools: COPE position statement. Available at: https://publicationethics.org/cope-position-statements/ai-author. Accessed 1 June 2023

[4] Jackson J, Landis G, Baskin PK, Hadsell KA, English M (2023) CSE guidance on machine learning and artificial intelligence tools. Sci Ed 46:72. 10.36591/SE-D-4602-07

[5] Lauer M, Constant S, Wernimont A (2023) Using AI in peer review is a breach of confidentiality. Available via: https://www.csr.nih.gov/reviewmatters/2023/06/23/usingai-in-peer-review-is-a-breach-of-confidentiality/

[6] Flanagin A, Bibbins-Domingo K, Berkwits M, Christiansen SL (2023). Nonhuman “authors” and implications for the integrity of scientific publication and medical knowledge. JAMA.

[7] Flanagin A, Kendall-Taylor J, Bibbins-Domingo K (2023). Guidance for authors, peer reviewers, and editors on use of AI, language models, and chatbots. JAMA.

[8] Writing the rules in AI-assisted writing (2023). Nat Mach Intell.

[9] Park SH (2023). Use of generative artificial intelligence, including large language models such as ChatGPT, in scientific publications: policies of KJR and prominent authorities. Korean J Radiol.

[10] Miller K, Gunn E, Cochran A (2023). Use of large language models and artificial intelligence tools in works submitted to Journal of Clinical Oncology. J Clin Oncol.

[11] American Association for the Advancement of Science. Science journals: editorial policies. Available at: https://www.science.org/content/page/science-journals-editorial-policies#authorship.

[12] Tejani A (2023) Large Language Models in medical journalism. Available via: https://pubs.rsna.org/page/ai/blog/2023/07/ryai_editorsblog070323

[13] Committee on Publication Ethics. Artificial intelligence (AI) in decision making. September 2021. 10.24318/9kvAgrnJ

